# Quadricuspid Aortic Valve Diagnosed by Cardiac CT

**DOI:** 10.1155/2011/572324

**Published:** 2011-07-11

**Authors:** Nisha D'Mello, Vikas Tandon, Benjamin J. W. Chow

**Affiliations:** Division of Cardiology, University of Ottawa Heart Institute, Ottawa, ON, Canada K1Y 1W2

## Abstract

Quadricuspid aortic valves are rare congenital anomalies which can be diagnosed by various imaging modalities. Described is the case of a 77 year old female with a quadricuspid aortic valve diagnosed by cardiac CT.

A 77-year-old woman was referred for the investigation of atypical chest pain and exertional dyspnea. Cardiac CT demonstrated the following: (1) a quadricuspid aortic valve (QAV) with four equally sized cusps, mildly thickened leaflets and incomplete coaptation (Figure 1, see Movie 1 in Supplementary Material available online at doi:10.1155/2011/572324), (2) a persistent left-sided superior vena cava and (3) juxtaposed left atrial appendage (Movie 2). 

Quadricuspid aortic valves are rare congenital anomalies with an incidence between 0.003 and 0.043% [1]. Embryologically, the aortic valve is formed when the truncus arteriosus separates into aortic and pulmonary trunks. Within the walls of the aortic and pulmonic trunks, three pads of mesenchymal tissue develop inward to form the primordia of the semilunar cusps. Abnormal cusp formation results from either aberrant fusion of the aorticopulmonary septum or from abnormal mesenchymal proliferation in the common trunk resulting in abnormal aortic valve cusps [2]. The most common QAV is characterized by 3 equal large cusps with one smaller cusp. 

Most QAVs are isolated congenital defects; however, various anomalies have been reported in association with QAV (patent ductus arteriosus, hypertrophic cardiomyopathy, subaortic stenosis, ventricular septal defect, and Ehlers-Danlos syndrome) [3]. As well, they can be associated with congenital coronary anomalies (single coronary ostium, displacement of the left and right coronary orifice) [4]. It is extremely important to delineate the coronary anatomy preoperatively to prevent ostial obstruction of the coronary artery during surgical implantation of a prosthetic ring. 

##  Conflict of Interests

The authors declared that there is no conflict of interests.

## Supplementary Material

Movie 1: Gated images of the short-axis view of a quadricuspid aortic valve demonstrating motion of four equally sized cusps with incomplete coaptation.
Movie 2: Axial images of the heart demonstrating a persistent left-sided superior vena cava and a left atrial appendage which is juxtaposed to the aorta.

## Figures and Tables

**Figure 1:**
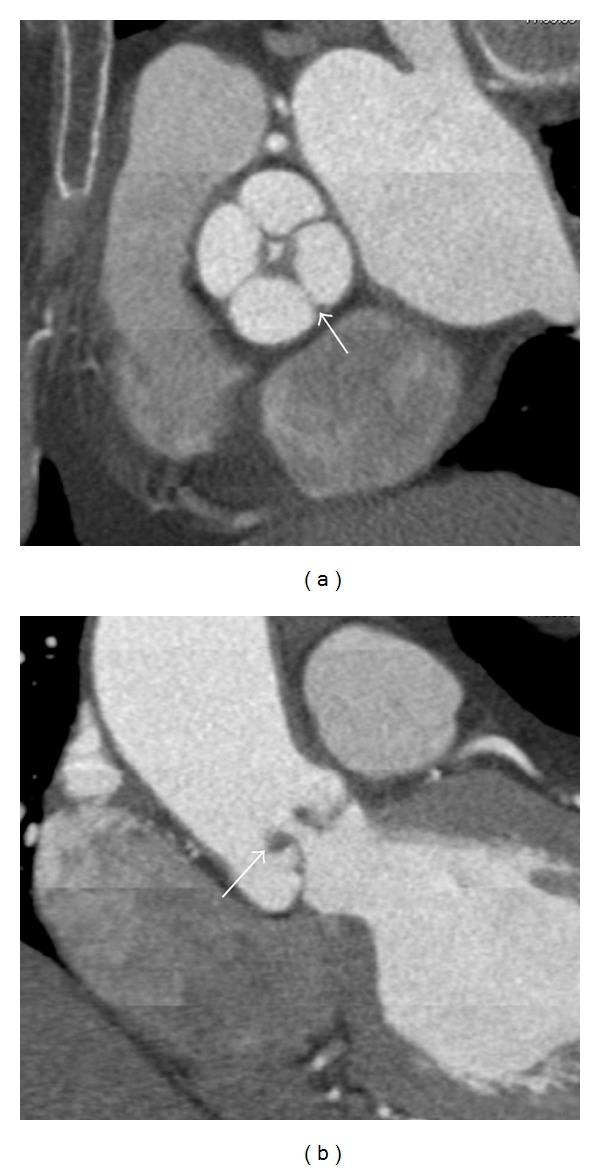
(a) Short-axis view of a quadricuspid aortic valve (arrow). (b) Long-axis view of a quadricuspid aortic valve with leaflet tip thickening (arrow).
